# Effect of temperature variation on hospital admissions and outcomes in dogs with myxomatous mitral valve disease and new onset pulmonary edema

**DOI:** 10.1371/journal.pone.0227807

**Published:** 2020-01-14

**Authors:** Carlo Guglielmini, Marco Baron Toaldo, Alex Chiesa, Barbara Contiero, Michele Berlanda, Helen Poser

**Affiliations:** 1 Department of Animal Medicine, Production and Health, University of Padua, Padua, Italy; 2 Department of Veterinary Medical Sciences, Alma Mater Studiorum—University of Bologna, Bologna, Italy; Michigan State University, UNITED STATES

## Abstract

The effect of seasonal variation on hospital admissions and outcomes in humans with cardiovascular disease and congestive heart failure (CHF) has been described. This study evaluates the effect of temperature variation on admissions and outcomes in dogs with myxomatous mitral valve disease (MMVD) and first onset CHF. Ninety-three client-owned dogs with MMVD and a first occurrence of pulmonary edema were included in this retrospective clinical cohort study. Recorded clinical and echocardiographic variables were accumulated and analyzed with dogs allocated into groups in a temperature-wise manner that considered the mean of the average (Tave) and maximum ambient temperature (Tmax) of the 14 days preceding hospital admission. A survival analysis was also performed. No difference was found in the percentage of dogs decompensating in three different temperature periods (i.e., cold, intermediate, and hot temperature) according to both Tave and Tmax. Dogs developing CHF during the intermediate temperatures according to Tmax died earlier from cardiac-related causes (median survival time 280 days, 95% CI = 147–486 days) compared to those decompensating during hot temperatures (median survival time 518 days, 95% CI = 344–819 days, *P* = 0.039). However, an effect of the ambient temperature on survival was not confirmed by Cox proportional hazard analysis. In conclusion, this study failed to show that ambient temperature has an effect on the first occurrence of CHF and outcomes in dogs with MMVD.

## Introduction

Myxomatous mitral valve disease (MMVD) is the most common canine cardiac disease, particularly in small-sized, aged dogs [1,2]. Degenerative valvular disease and associated mitral regurgitation can lead to left-sided cardiac dilation and dysfunction and eventually to overt congestive heart failure (CHF), most commonly manifesting as cardiogenic pulmonary edema. In small breed dogs, MMVD is one of the most frequent causes of death overall [3]. Various prognostic indicators have been investigated in dogs with MMVD, in particular using various echocardiographic techniques, and different clinical variables associated with cardiac mortality have been identified [4–18]. Among them, breed of dog, advanced age, and body weight (BW) ≥ 20 kg, as well as degree of mitral valve pathology; left atrial (LA) and left ventricular (LV) dilation and dysfunction; increased LA pressure; right ventricular dysfunction; development of atrial fibrillation (AF) and pulmonary hypertension (PH); and increased concentration of circulating cardiac biomarkers (e.g., NT-pro-BNP) have been associated with a worse prognosis [4–18]. On the contrary, use of several drugs, including ACE-inhibitors, pimobendan, and spironolactone has a proven positive effect both on the quality and duration of life in dogs with MMVD [19–24].

In humans, the effect of seasonal variation on CHF-related hospitalization, as well as the clinical outcomes in patients admitted for CHF has been investigated in different studies [25–30]. In particular, an effect of ambient temperature has been described with a winter peak for many cardiovascular events including CHF while conflicting results have been reported for successive outcomes [25–30]. To the best of our knowledge, only one study evaluated the circadian and seasonal presentations of 119 dogs with CHF caused by various cardiovascular diseases [31]. However, no systematic study has specifically addressed the effect of temperature variation on hospital admissions and outcomes in dogs with MMVD and CHF. Therefore, the aims of the present study are: (1) to investigate the effect of ambient temperature on the admission of dogs with MMVD at the time of the first occurrence of cardiogenic pulmonary edema; and (2) to evaluate if the temperature at admission, in addition to other previously recognized variables, could have a prognostic value in dogs with MMVD.

## Materials and methods

### Animals

This is a retrospective study based on the retrieval of clinical data of dogs with MMVD. All the information included in the study was obtained following standardized diagnostic techniques for this type of animal. Therefore, no specific approval was requested and obtained, but all the owners of the enrolled dogs signed a written consent, which considered the diagnostic procedures and the eventual use of the obtained data for research purposes. Medical records of all dogs with MMVD at the time of first occurrence of cardiogenic pulmonary edema at two Italian Veterinary Teaching Hospitals (VTHs) from January 2011 and December 2016 (corresponding to a total of 2192 days) were retrospectively reviewed. Diagnosis was based on combined clinical, electrocardiographic, radiographic, and echocardiographic findings. In particular, clinical signs included tachypnea and/or dyspnea, tachycardia, systolic heart murmur, and abnormal lung sounds; radiographic findings included increased pulmonary opacity resulting from unstructured interstitial or mixed interstitial-alveolar pattern associated with enlarged cardiac silhouette [32,33]. Transthoracic echocardiography, two-dimensional (2D), M-mode, and echo-Doppler, was performed by one experienced operator at each center (HP and MBT) within a maximum of 48 h from the radiographic examination using one of several ultrasound units **(**Zone Ultra, Zonare Medical Systems, Mountain View, CA; CX50, Philips, Eindhoven, Netherlands; iU22 ultrasound system, Philips Healthcare, Monza, Italy; iE33 ultrasound system, Philips Healthcare, Monza, Italy) equipped with phased array transducers and continuous ECG tracing. Dogs with echocardiographic findings typical for MMVD (i.e., thickened and/or prolapsing mitral valve leaflets, mitral regurgitation on color flow Doppler) and without congenital cardiac malformations or other acquired cardiac diseases were included. Additional clinical information included type of cardiac rhythm (e.g., presence or absence of AF) and previous use of any cardiovascular drugs.

After diagnosis of cardiogenic pulmonary edema, all dogs were treated with furosemide, an ACE-inhibitor, and pimobendan; eight dogs also received spironolactone, two of which also received amiodarone.

### Echocardiographic examination

Left ventricular (LV) internal diameters were measured from 2D guided M-mode images obtained from a right parasternal short axis view at the level of chordae tendinae. The diameters of the LV were then normalized for the effect of the BW using the following equations: normalized LV diastolic diameter (LVDDn) = LV diastolic diameter/[BW^0.294^] and normalized LV systolic diameter (LVSDn) = LV systolic diameter/[BW^0.315^], as previously described [34]. The LA and aortic (Ao) diameter was measured at early diastole (i.e., the first frame following aortic valve closure) on 2D images obtained from a right parasternal short axis view at the level of the Ao root and the LA:Ao was calculated [35]. Trans-mitral diastolic inflow was recorded from the left parasternal apical 4 chamber view with the sample volume positioned at the level of the mitral valve tips and the peak velocity of early diastolic blood flow (E-max) was measured. Three consecutive measurements were averaged for each echocardiographic and echo-Doppler variable.

The presence of PH was considered for dogs without right ventricular outflow obstruction and measurable tricuspid regurgitation (TR) with a peak velocity ≥ 2.8 m/s, corresponding to a peak tricuspid regurgitation pressure gradient ≥ 31 mmHg [36]. In dogs without TR, the PH status could not be determined.

### Temperature assessment

The two VTHs where the study was carried out are located in the Northern hemisphere in a region characterized by continental climate with harsh winters and hot summers. For each dog, the day of first occurrence of cardiogenic pulmonary edema was retrieved at each center. A search was then made on the websites of the Regional Agency for Environmental Prevention and Protection (Agenzia Regionale per la Prevenzione e la Protezione Ambientale Veneto: http://www.arpa.veneto.it/ and Agenzia Regionale per la Prevenzione e la Protezione Ambientale Emilia Romagna https://www.arpae.it/, accessed 24–27 July 2017) for the average (i.e., the average temperature over a 24-hour period, Tave) and maximum ambient temperature (Tmax) for the 14 days prior to the development of pulmonary edema. The means for Tave and Tmax were then calculated over this 14 day period (i.e., the sum of the Tmax for the preceding 14 days divided by 14) in the location where decompensation occurred (i.e., the location of the two VTHs). After calculating the mean values of these temperatures, six overall different temperature ranges were considered, three for Tave and three for Tmax: cold temperature, when the Tave or Tmax was ≤ 10°C (grossly corresponding to a winter admission); intermediate temperature, when the Tave or Tmax was > 10°C and ≤ 20°C (grossly corresponding to a spring-autumn admission); and hot temperature, when the Tave or Tmax temperature was > 20°C (grossly corresponding to a summer admission). The number of days belonging to these six temperature periods (three for Tave and three for Tmax) was also calculated. Dogs with MMVD were then divided into groups according to the classification of temperature at the time they presented with a first episode of CHF. Finally, the number of dogs in each of the six considered temperature periods was divided by the number of days in the same temperature period and multiplied by 100 to obtain the percentage of dogs presented for pulmonary edema in each considered temperature period.

### Statistical and survival analysis

Normal distribution of clinical, echocardiographic, and echo-Doppler variables was assessed using a Shapiro-Wilk test. Normally distributed data were reported as mean ± standard deviation and data with nonparametric distribution were expressed as median and range. The one-way ANOVA and the Kruskal-Wallis test were used to compare data of normally and non-normally distributed variables, respectively, among dogs distributed within the three groups according to the Tave or Tmax. For the nominal data type of breed (i.e., purebred vs mixed breed), sex, presence of AF and PH, and previous use of cardiovascular drugs, differences were evaluated by the Chi-square test. This test was also used to compare the percentage of dogs decompensating in each of the three different temperature periods according to Tave or Tmax. A post hoc test with a Bonferroni correction was applied when the factors were significant.

Survival data were obtained from internal databases or through telephone interviews with the referring veterinarian or owner. Dogs were classified as still alive, dead from cardiac related causes (defined as due to sudden death, CHF refractory to medical therapy, euthanasia due to worsening of the cardiac condition, or death within four hours from the diagnosis of pulmonary edema), dead from cardiac unrelated causes, or lost to follow up when no further data were available after presentation [4]. Time in days from the diagnosis of pulmonary edema and CHF to death (survival time) or to the telephone call for dogs still alive (follow-up time) was recorded. Dogs still alive at the end of the observational period and dogs that died for non-cardiac related events were right-censored. The Kaplan-Meier survival analysis was employed to evaluate the effect of Tave and Tmax on survival time. Pairwise post-hoc log-rank analyses with Bonferroni correction were then applied.

Cox proportional univariate regression analysis was performed to determine whether a significant relationship existed between clinical variables, as well as Tave and Tmax considered as continuous variables, and survival endpoint. The hazard ratio (HR) and 95% confidence intervals (CI) were calculated considering one-unit change for the continuous variables. Variables with P < 0.1 in the univariate analysis were entered into a multivariable Cox proportional hazard analysis using a manual forward selection method to identify independent predictors of survival.

Because of the retrospective design of this study there is a limited control of the number of observations. Thus, a power analysis was finally performed on available data to calculate the power of the study.

All statistical analyses were performed using commercially available statistical software programs (SAS version 9.3, SAS Institute Inc., Cary, NC, USA; MedCalc version 12.6.1.0, MedCalc Software, Ostend, Belgium). The level of significance was set at P < 0.05.

## Results

### Study population

Ninety-three dogs (55.9% male and 44.1% female) met the inclusion criteria and follow up data were available for 86 (92.5%) of them. These dogs belonged to 17 different breeds, with mixed breed being the most frequently presented group (50.5%), followed by Cavalier King Charles Spaniel (7.5%), Dachshund and Maltese (6.5% each), and Miniature Poodle (5.4%). Their mean age was 11.2 ± 2.7 years and the BW was 7.9 ± 3.5 kg. Thirty-one dogs (33.3%) were receiving some cardiovascular therapy before the onset of CHF, including 17 and 14 dogs that received one or two cardiovascular drugs, respectively. Seventy-two dogs had recordable TR (77.4%) and PH was diagnosed in 44 of these (47.3%). In the remaining 21 dogs (22.6%) no TR or pulmonic insufficiency was found and, therefore, the pulmonary pressure status could not be assessed. Atrial fibrillation was diagnosed in two dogs (2.2%). Tables 1 and 2 summarize the descriptive statistics for clinical and echocardiographic variables of all dogs.

**Table 1:** Clinical data in 93 dogs with decompensated myxomatous mitral valve disease according to the mean of the environmental average temperature (Tave) in the 14 days preceding the first onset of cardiogenic pulmonary edema.

Variable	Cold Temperature (Tave ≤ 10°C)	Intermediate Temperature (10°< Tave≤ 20°C)	Hot Temperature (Tave> 20°C)	Total	P-value
Dogs (N [%])	34 (36.6)	31 (33.3)	28 (30.1)	93 (100)	NA
Days (N [%])	765 (34.9)	816 (37.2)	611 (27.9)	2192 (100)	NA
Dogs/Days (%)	4.4	3.8	4.6	4.2	0.724
Age (years)	11.0 ± 2.8	11.1 ± 2.8	11.5 ± 2.5	11.2 ± 2.7	0.759
Body weight (kg)	7.4 ± 3.6	8.8 ± 3.9	7.6 ± 2.8	7.9 ± 3.5	0.227
Sex (male/female)	20/14	18/13	14/14	52/41	0.751
Breed (PB/MB)	15/19	16/15	15/13	46/47	0.728
LA:Ao	2.46 ± 0.57	2.42 ± 0.53	2.55 ± 0.50	2.5 ± 0.5	0.655
LVDDn	2.18 ± 0.35	2.13 ± 0.37	2.05 ± 0.28	2.13 ± 0.34	0.345
LVSDn	1.11 ± 0.25	1.12 ± 0.27	1.05 ± 0.26	1.09 ± 0.26	0.536
E-max (m/s)	1.46 ± 0.39	1.33 ± 0.32	1.30 ± 0.35	1.36 ± 0.36	0.177
PH (N [%])	16 (47.1)	13 (41.9)	15 (53.6)	44 (47.3)	0.670
AF (N [%])	1 (2.9)	1 (3.2)	0 (0)	2 (2.2)	0.642
Therapy (N [%])	9 (26.5)	12 (38.7)	10 (35.7)	31 (33.3)	0.550
ACE-I (N [%])	3 (8.8)	3 (9.7)	2 (7.1)	8 (8.6)	0.940
Furo (N [%])	2 (5.9)	4 (12.9)	2 (7.1)	8 (8.6)	0.570
Pimo (N [%])	1 (2.9)	0 (0)	0 (0)	1 (1.1)	0.416
ACE-I + Pimo (N [%])	0 (0)	0 (0)	2 (7.1)	2 (2.2)	0.591
ACE-I + Furo (N [%])	3 (8.8)	3 (9.7)	3 (10.7)	9 (9.7)	0.969
Furo + Pimo (N [%])	0 (0)	2 (6.5)	1 (3.6)	3 (32.2)	0.372

Data are expressed as mean ± SD or median (min-max).

Abbreviations: N, Number; PB, purebred; MB, mixed breed; LA:Ao, left atrium to aorta ratio; LVDDn, Left ventricular diastolic diameter normalized for body weight; LVSDn, Left ventricular systolic diameter normalized for body weight; E-max, Trans-mitral peak E-wave velocity; PH, Pulmonary hypertension; AF, Atrial fibrillation; ACE-I, angiotensin converting enzyme inhibitor; Furo, Furosemide; Pimo, Pimobendan; NA, not applicable.

**Table 2:** Clinical data in 93 dogs with decompensated myxomatous mitral valve disease according to the mean of the environmental maximum temperature (Tmax) in the 14 days preceding the first onset of cardiogenic pulmonary edema.

Variable	Cold Temperature (Tmax ≤ 10°C)	Intermediate Temperature (10°< Tmax≤ 20°C)	Hot Temperature (Tmax> 20°C)	Total	P-value
Dogs (N [%])	21 (22.6)	30 (32.2)	42 (45.2)	93 (100)	NA
Days (N [%])	414 (18.9)	751 (34.2)	1027 (46.9)	2192 (100)	NA
Dogs/Days (%)	5.1	4.0	4.1	4.2	0.646
Age (years)	11.4 ± 2.4	10.8 ± 3.0	11.4 ± 2.6	11.2 ± 2.7	0.629
Body weight (kg)	7.3 ± 4.2	8.4 ± 3.0	7.9 ± 3.5	7.9 ± 3.5	0.538
Sex (male/female)	12/9	19/11	21/21	52/41	0.528
Breed (PB/MB)	9/12	13/17	24/18	46/47	0.405
LA:Ao	2.49 ± 0.56	2.44 ± 0.57	2.55 ± 0.50	2.5 ± 0.5	0.910
LVDDn	2.20 ± 0.22	2.15 ± 0.45	2.05 ± 0.28	2.13 ± 0.34	0.357
LVSDn	1.11 ± 0.20	1.14 ± 0.32	1.05 ± 0.26	1.10 ± 0.26	0.349
E-max (m/s)	1.44 ± 0.36	1.39 ± 0.39	1.30 ± 0.36	1.37 ± 0.36	0.407
PH (N [%])	10 47.6)	15 (50)	19 (45.2)	44 (47.3)	0.923
AF (N [%])	1 (4.8)	1 (3.3)	0 (0)	2 (2.2)	0.406
Therapy (N [%])	7 (33.3)	9 (30.0)	15 (35.7)	31 (33.3)	0.879
ACE-I (N [%])	1 (4.8)	4 (13.3)	3 (7.1)	8 (8.6)	0.506
Furo (N [%])	1 (4.8)	3 (10.0)	4 (9.5)	8 (8.6)	0.773
Pimo (N [%])	1 (4.8)	0 (0)	0 (0)	1 (1.1)	0.177
ACE-I + Pimo (N [%])	1 (4.8)	0 (0)	1 (2.4)	2 (2.2)	0.509
ACE-I + Furo (N [%])	3 (14.3)	0 (0)	6 (14.3)	9 (9.7)	0.093
Furo + Pimo (N [%])	0 (0)	2 (6.7)	1 (2.4)	3 (32.2)	0.260

Data are expressed as mean ± SD or median (min-max).

Abbreviations: N, Number; PB, purebred; MB, mixed breed; LA:Ao, left atrium to aorta ratio; LVDDn, Left ventricular diastolic diameter normalized for body weight; LVSDn, Left ventricular systolic diameter normalized for body weight; E-max, Trans-mitral peak E-wave velocity; PH, Pulmonary hypertension; AF, Atrial fibrillation. ACE-I, angiotensin converting enzyme inhibitor; Furo, Furosemide; Pimo, Pimobendan; NA, not applicable.

### Temperature assessment and survival analysis

On the total of 2192 days included in the observation period, there were 765 days (34.9%) 816 days (37.2%), and 611 days (27.9%) with cold, intermediate, and hot temperature, respectively, according to Tave and 34 dogs (36.6%), 31 dogs (33.3%), and 28 dogs (30.1%) developed CHF during the cold, intermediate, and hot temperature period, respectively. No difference was found in the percentage of dogs with a first episode of CHF in each corresponding temperature period (P = 0.724). Furthermore, no difference was found for all the considered clinical and echocardiographic variables among these three groups of dogs (Table 1).

According to the Tmax, there were 414 days (18.9%), 751 days (34.2%), and 1027 days (46.9%) with cold, intermediate, and hot temperature, respectively and 21 dogs (22.6%), 30 dogs (32.2%), and 42 dogs (45.2%) developed CHF during the cold, intermediate, and hot temperature period, respectively. Similar to that observed for Tave, no difference was found for either the percentage of dogs with a first episode of CHF in each temperature range (P = 0.646) or for any of the considered clinical and echocardiographic variables among these three groups of dogs (Table 2).

**Fig 1:**
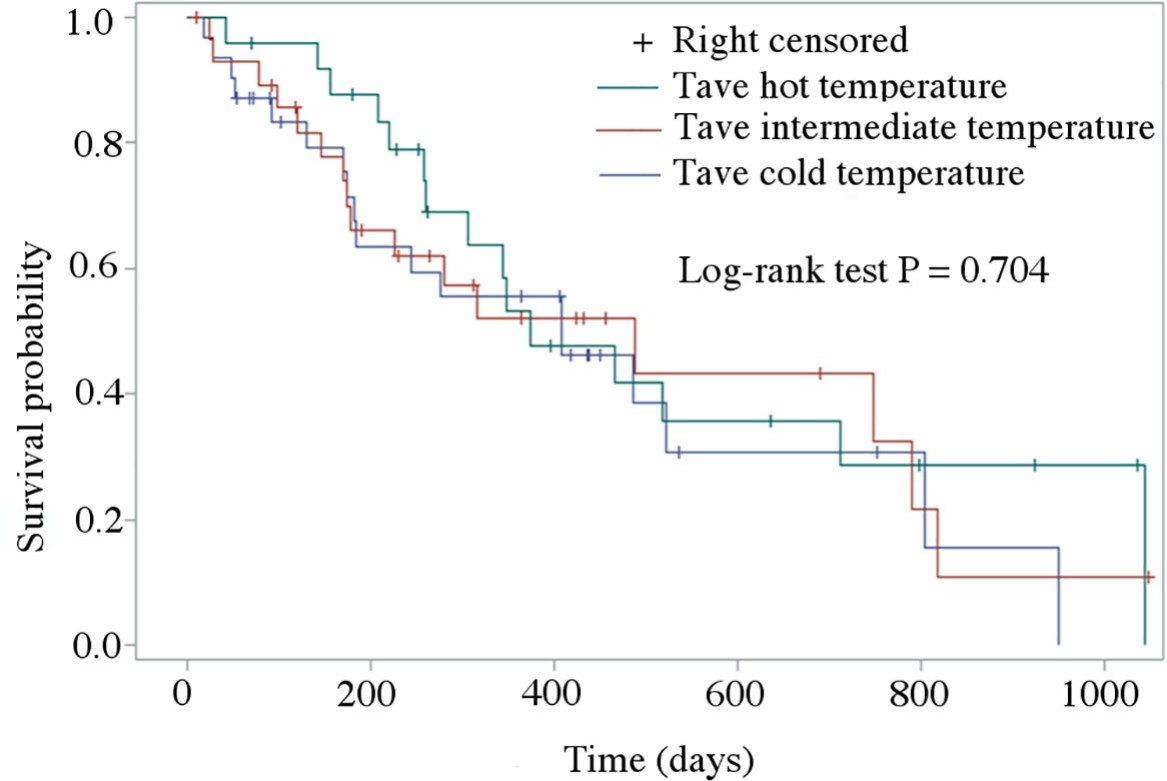
Kaplan-Meier survival curves of dogs divided based on the average ambient temperature (Tave). Dogs still alive at the end of the observational period and dogs that died for non-cardiac related events were right-censored. Cold temperature (blue line): Tave **≤** 10°C; Intermediate temperature (red line): Tave >10°C and **≤** 20°C; Hot temperature (green line): Tave > 20°C. No difference was found among groups (log-rank test P = 0.704).

**Fig 2:**
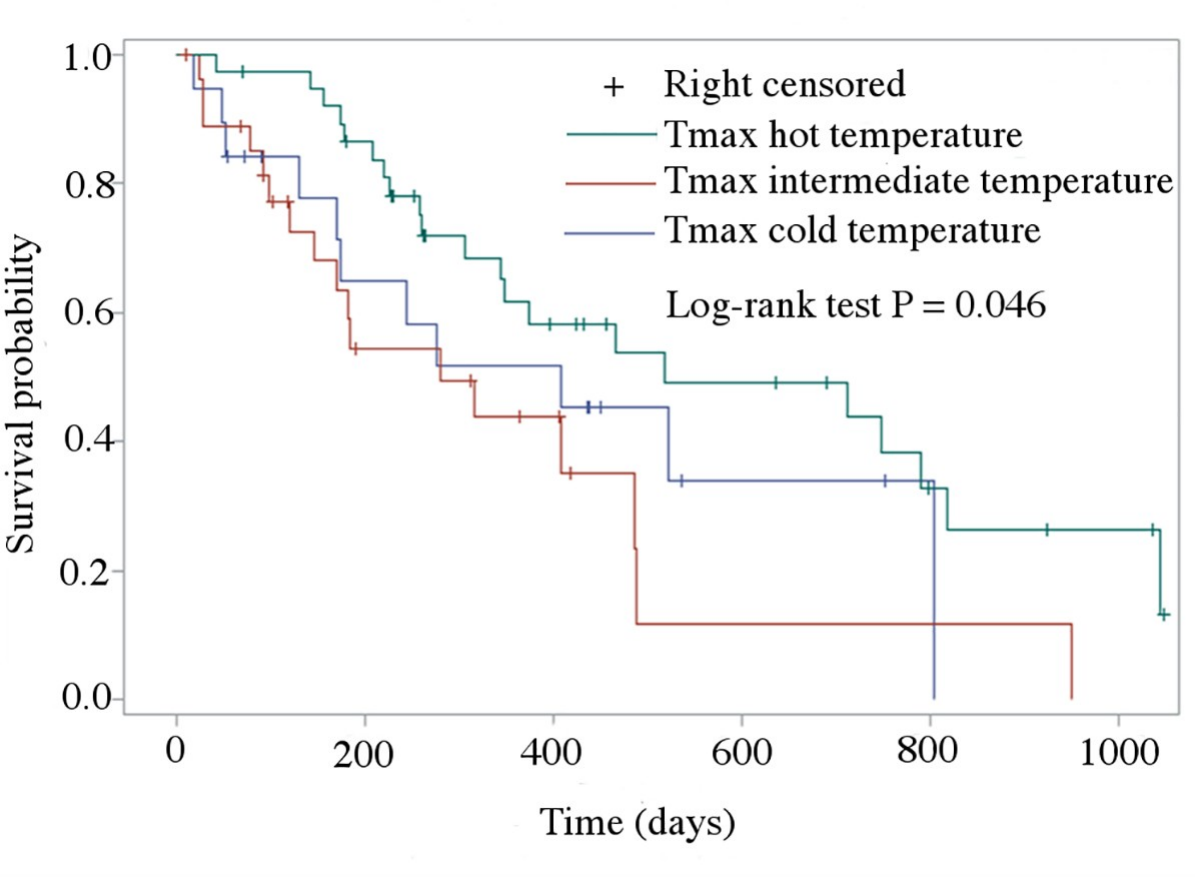
Kaplan-Meier survival curves of dogs divided based on the average of the maximal ambient temperature (Tmax). Dogs still alive at the end of the observational period and dogs that died for non-cardiac related events were right-censored. Dogs decompensating during the hot temperature had a significantly longer median time to death for a cardiac related cause (518 days, 95% CI = 344–819 days) compared to those decompensating during the intermediate climate (280 days, 95% CI: 147–486 days; P = 0.039). Cold temperature (blue line): Tave **≤** 10°C; Intermediate temperature (red line): Tave >10°C and **≤** 20°C; Hot temperature (green line): Tave > 20°C.

At the end of the follow up period, 49 dogs had died due to cardiac-related causes (median survival time, 227 days; range, 19–1044 days), 11 dogs had died of non-cardiac-related causes (median survival time, 425 days; range, 90–925 days), 26 dogs were still alive (median follow-up time, 289 days; range, 10–1048 days), while seven dogs were lost to follow-up. Figs 1 and 2 show the Kaplan Meier survival curve comparing dogs developing CHF in the three considered temperature periods according to Tave and Tmax, respectively. Based on this analysis, calculated accounting for censored data, no difference was found in dogs decompensating during the different temperature period according to Tave (log-rank test P = 0.704), whereas a difference was found according to Tmax (log-rank test P = 0.046). In particular, the 17 dogs with follow up available presented with CHF during the intermediate temperature according to Tmax died earlier for a cardiac-related cause (median time to death 280 days, 95% CI = 147–486 days) than the 21 dogs presented with CHF during the hot temperature (median time to death 518 days, 95% CI = 344–819 days, P = 0.039).

The univariate analysis of 12 covariables (age, BW, sex, breed, PH, AF, LA:Ao, LVDDn, LVSDn, Emax, Tave, and Tmax) for their association to the time to endpoint showed that only PH, AF, LVDDn, and Emax had a P value < 0.1 (Table 3), whereas neither Tave nor Tmax were associated with a positive or negative clinical outcome (HR = 0.978 and 0.978, 95% CI = 0.944–1.012 and 0.948–1.010, P = 0.207 and 0.176, respectively). When the above four variables were entered into the multivariable analysis, only PH (HR = 2.039, 95% CI = 1.003–4.148, P = 0.045) and AF (HR = 20.561, 95% CI = 3.724–113.525, P < 0.001) were retained (Table 4).

**Table 3:** Cox proportional hazard univariate analysis in dogs with decompensated myxomatous mitral valve disease.

Predictors	HR	95%CI	*P*-value
Age (year)	1.001	0.903–1.111	0.979
BW (kg)	0.987	0.909–1.071	0.752
Sex (male/female)	1.002	0.568–1.767	0.994
Breed (PB/MB)	1.456	0.823–2.576	0.197
LA:Ao	1.565	0.879–2.785	0.128
LVDDn	2.515	0.898–7.044	0.079
LVSDn	2.496	0.678–9.196	0.169
E-max (m/s)	2.419	1.040–5.627	0.040
PH	1.776	0.911–3.462	0.092
AF	25.081	4.785–131.451	<0.001
Tave (°)	0.978	0.944–1.012	0.207
Tmax (°)	0.978	0.948–1.01	0.176

HR = hazard ratio; CI, confidence interval; BW, body weight; PB, purebred; MB, mixed breed; LA:Ao, left atrium to aorta ratio; LVDDn, Left ventricular diastolic diameter normalized for body weight; LVSDn, Left ventricular systolic diameter normalized for body weight; E-max, Trans-mitral peak E-wave velocity; PH, Pulmonary hypertension; AF, Atrial Fibrillation; Tave, average temperature; Tmax, maximum temperature.

**Table 4:** Final model results in the multivariable Cox proportional hazard analysis in dogs with decompensated myxomatous mitral valve disease.

Predictors	HR	95%CI	*P*-value
PH	2.039	1.003–4.148	0.045
AF	20.561	3.724–113.525	< 0.001

HR = hazard ratio; CI, confidence interval; PH, Pulmonary hypertension; AF, Atrial Fibrillation.

For the power analysis, the highest and lowest median survival time recorded for dogs presented with pulmonary edema during the hot and intermediate temperature according to Tmax, (518 days and 280 days, respectively), were used with a variability of 400 days calculated on the basis of the standard error produced by the survival analysis. Considering the sample size of each group (42 dogs and 30 dogs, respectively) the calculated power of the test was 69%. If less exceptional results had been considered, for example by comparing data obtained during hot and cold temperature according to Tmax, a lower power would have been obtained. Seventy-nine animals in each temperature group would have an 80% power for detecting a 64% difference in the median survival time with a P value < 0.05.

## Discussion

In the present study, we found a shorter survival time for dogs with MMVD developing CHF during the intermediate temperature according to Tmax. However, our study failed to demonstrate that ambient temperature has an effect hospital admission and outcome in these dogs. To study the effect of the ambient temperature on the first occurrence of CHF in dogs with MMVD, we considered the precise measurement of atmospheric temperatures in the 14 days preceding the decompensation rather than considering the simple seasonal or monthly trends, as previously performed in some humans and veterinary studies [25–31]. This approach allowed us to overcome any variations in ambient temperature related to different seasonal trends (e.g., hotter or cooler summer in successive years) and to evaluate the actual chronobiological effect of the ambient temperature on the natural history of canine MMVD. Furthermore, the limitation associated with the difficulty in establishing the time difference between onset of symptoms of pulmonary edema and when the owner brought the animal to the VTH was likely overcome using a 2-week time of temperature observation [31].

No difference was found in any of the considered clinical and echocardiographic variables after dividing dogs of the present study according to the Tave and Tmax in the days preceding the onset of pulmonary edema. These variables included those indicating cardiac remodeling (i.e., LA:Ao, LVDDn, LVSDn), increased LA pressure (i.e., E-max), and comorbidities associated with worsening of valvular disease (i.e., presence of PH and AF) and all had shown a negative prognostic effect in dogs with MMVD [4–14]. Furthermore, no difference was found in either the percentage of dogs on treatment or in the type of cardiovascular drugs employed prior to CHF development. These findings indicate that dogs with MMVD and CHF divided in the temperature-based groups did not have any difference in the severity of their valvular disease.

No difference was found in the percentages of dogs presented for first occurrence pulmonary edema according to the three temperature periods for either Tave or Tmax. These findings suggest that the observed incidence of CHF was not different to what would be expected. In a previous study, the fall months September and October had the highest number of hospital admissions of 119 dogs with acute presentation of CHF caused by different cardiac diseases [31]. However, a comparison with results of the present study is difficult because that study included a heterogeneous population of dogs with different cardiac diseases and extrapolation of data regarding only those dogs with MMVD is impossible. In people, a seasonal or monthly pattern of CHF presentations has been described and different studies reported that the peak of admission for CHF was the winter season [25–30]. In addition to pathophysiological changes associated with temperature reductions (e.g., increased catecholamine release and sympathetic surge leading to increase vascular tone and heart rate, high blood pressure, and cardiovascular event) seasonal variation of CHF-related comorbidities including respiratory tract infections (particularly influenza and pneumonia), myocardial ischemia and infarction, systemic hypertension, and cardiac arrhythmias are considered responsible for higher numbers of human hospitalization during cold climate [26–28]. The information regarding chronobiologic changes of sympathetic tone and comorbidities influencing hospitalization in dogs with cardiac disease is lacking. However, with the only exception of AF [14] the above listed comorbidities reported for human patients can be considered unlikely in canine MMVD and no chronobiological effect has been reported for other potentially concomitant canine airway diseases such as tracheal collapse, bronchomalacia, and chronic bronchitis [32,37–39].

Conflicting results have been reported in the human literature regarding the effect of seasonal variation in hospitalization outcomes of patients admitted for CHF. A negative impact of the summer season has been described in some studies [26,28], while worse hospitalization outcomes for winter admission has been reported in another more recent study [30]. However, seasonal associated differences in the clinical condition of patients hospitalized for CHF has been acknowledged and a more critical clinical condition, as well as a more advanced age in the summer admission has been reported [26,28]. These differences in clinical condition at admission can bias the observations on successive outcome. Results of the present study are likely unbiased by seasonal differences in clinical condition at admission since no difference was found for the considered clinical variables among the different groups of dogs. Although, a longer survival time for cardiac related death was found in dogs with first occurring pulmonary edema during the hot temperature compared to those first presented during the intermediate temperature according to Tmax, these results were not confirmed by the Cox proportional hazard analysis. In particular, the univariate analysis identified increased E-max and LV diastolic dimension, and development of AF and PH as variables potentially associated with a negative prognosis, whereas only AF and PH were retained following the multivariable analysis. The observed discrepancy between the results of the Kaplan Meyer analysis and those of Cox proportional hazard analysis is likely associated with the different use of the variables Tave and Tmax in the two statistical analyses. In the Kaplan Meyer analysis, they were treated as categorical variables while in the Cox proportional hazard analysis they were treated as continuous variables. Surprisingly, some previously demonstrated strong risk factors for cardiac death in dogs with decompensated MMVD (e.g., LA:Ao and increased LV dimension [23]) were not confirmed as negative predictors in this study. The lower number of dogs included in the present study and the more homogeneous distribution of data are likely responsible for these different results.

This study has several limitations because of its retrospective design. First, since very few significant differences were found, it is possible that the study was not adequately powered to show a difference that actually exists among the considered variables. Thus, the presence of a type II statistical error could not be completely excluded. Second, data were collected from two VTHs located in the same geographical and climatic area of the Northern hemisphere, about 100 km away from each other. Therefore, the results of this study might be valid for other veterinary centers located in geographic areas with a similar climate but cannot be generalized to all veterinary centers. Third, other climatic factors, including relative humidity, atmospheric pressure, and air pollutant exposure [40], or patient-related factors, including percentage of time the dog spent indoor/outdoor and access to indoor climate control (i.e., heating and air-conditioning), not measured in this study can have an effect on development and outcome of CHF. Finally, the ambient temperature data were obtained from the VTH location from the website. Thus, they might not reflect the actual temperature of the place where animals developed HF. However, the admission of dogs with MMVD and first occurrence of pulmonary edema coming from distant areas with different climate and ambient temperature is unlikely.

## Conclusions

In conclusion, the results of the present study failed to demonstrate that ambient temperature, measured both as Tmax and Tave, in the preceding 14 days, has a significant effect on the first occurrence of pulmonary edema in dogs with MMVD. The ambient temperature did not even show a significant effect on CHF outcomes in the same animals, differently from what has been reported in humans. The absence of comorbidities affecting human patients with CHF is likely responsible for these different results between humans and dogs although the low number of dogs included in the study could be also responsible of this negative result.

## Supporting information

S1 Dataset (XLSX)
