# Correction: Effect of temperature variation on hospital admissions and outcomes in dogs with myxomatous mitral valve disease and new onset pulmonary edema

**DOI:** 10.1371/journal.pone.0250520

**Published:** 2021-04-15

**Authors:** Carlo Guglielmini, Marco Baron Toaldo, Alex Chiesa, Barbara Contiero, Michele Berlanda, Helen Poser

The second author’s name appears incorrectly in the citation. The correct citation is: Guglielmini C, Baron Toaldo M, Chiesa A, Contiero B, Berlanda M, Poser H (2020) Effect of temperature variation on hospital admissions and outcomes in dogs with myxomatous mitral valve disease and new onset pulmonary edema. PLoS ONE 15(1): e0227807. https://doi.org/10.1371/journal.pone.0227807.
